# Incidental Findings of Asymptomatic Fungal Infection

**DOI:** 10.1155/2022/3694968

**Published:** 2022-09-05

**Authors:** Nisha Manila, Madhu Nair, Hui Liang

**Affiliations:** ^1^Oral and Maxillofacial Radiology, Department of Diagnostic Sciences, Louisiana State University School of Dentistry, New Orleans, Louisiana, USA; ^2^Oral and Maxillofacial Radiology, Director of Imaging Center, Texas A&M College of Dentistry, Dallas, Texas, USA; ^3^Director of Oral and Maxillofacial Radiology Resident Program, Department of Diagnostic Sciences, Texas A&M College of Dentistry, Dallas Texas, USA

## Abstract

Fungal sinusitis of the paranasal sinuses is a rare infection in healthy individuals but is relatively common in immunocompromised patients. It is often misdiagnosed and frequently a severe disease, as a few forms of it are linked with a higher mortality rate. Effective handling necessitates a speedy analysis and often counts on radiological findings. On cone-beam computed tomography (CBCT) analysis, a bulky polypoid-shaped lesion with a density close to that of soft tissue in CBCT was visualized in the right ethmoid and sphenoid sinuses. There was a significant expansion of the borders of the right ethmoid sinus, and discontinuity or perforation of the sphenoid sinus floor was suspected from CBCT images. Non-contrasted multi-detector computed tomography (MDCT) exhibited opacification and extension of the lesion into the majority of sinuses with dense inspissated materials in the center, which resembled radiographic features of invasive fungal sinusitis. Computed tomography (CT) scan of the maxillofacial region, specifically paranasal sinuses, plays a considerable role in diagnosing fungal sinusitis. In a majority of cases, fungal sinusitis is noticed and diagnosed in immunocompromised patients. However, it is also seen in healthy patients in very rare circumstances, similar to the patient in this report. If the patient is treated rapidly, the prognosis is fair. We present a case of fungal sinusitis in an otherwise healthy young male patient to increase awareness among dental professionals.

## 1. Introduction

Fungal sinusitis or pansinusitis is considered an uncommon inflammatory process of the paranasal sinuses, and it primarily affects immunocompromised patients and occasionally immunocompetent individuals. The recent increase in the reporting of this condition is attributed to advanced diagnostic techniques, immunodeficiency caused by cancer treatment, diabetes, overuse of antibiotics, and post-transplant therapy [1].

Fungal infection of the paranasal sinuses is broadly classified as noninvasive or invasive based on the non-appearance or appearance of the fungal hyphae in tissue, submucosa, mucosa, blood, or bone of the paranasal sinus. Noninvasive infection is further sub-categorized as allergic fungal sinusitis and fungal mycetoma (fungal ball). Invasive type is further sub-divided into acute, chronic, and granulomatous invasive fungal sinusitis [2]. A retrospective audit study conducted between January 2000 and August 2007 revealed that 63% were affected by allergic fungal sinusitis, and 34% had an invasive form of fungal infection. *Rhizopus arrhizus* remained the primary causative agent of acute invasive sinusitis, whereas *Aspergillus fumigates* was the primary contributing factor for allergic fungal sinusitis [3].

The acute invasive form is an aggressive infection observed mainly in poor diabetic control and immunocompromised patients. It reports a mortality rate of 50–80%, making it the most lethal variant [2]. To differentiate between the two primary forms of fungal sinusitis, it is integral to (a) collect appropriate amounts of exudates and other contents from sinuses, (b) biopsy samples of unaffected and affected mucosa, and (c) biopsy the bone adjacent to parts of necrosis [2]. This type of fungal infection cannot be stained with regular stains. Distinct silver-infused fungal dyes and fungal cultures are mandatory for explicit confirmation and further sub-classification of this condition. Effective treatment requires accurate and early investigation, which frequently depends on diagnostic imaging, precisely computed tomography (CT) and magnetic resonance (MR) imaging. CT evaluates bone changes accurately, and MR imaging identifies intraorbital and intracranial extensions. The prevalent knowledge indicates that invasive variant is often seen in immunodeficient patients, whereas noninvasive primarily occurs in immunocompetent individuals [4]. The treatment strategies vary for each subtype, and so as their prognoses. It is crucial to have a deeper insight into the various forms of fungal infections and their radiographic manifestations. These diagnostic imaging features are the red flag alerts that help the radiologist notify the clinician. It will also support the use of appropriate final diagnostic techniques for confirmation of the disease and help play an integral role in patient care. Apt diagnosis and instigation of proper care are needed to avoid an undesirable fatal outcome.

We report an incidental finding of fungal sinusitis in a healthy 21-year Caucasian male, contrary to the usual findings. We aim to highlight the role played by radiologists in the early detection of this case and emphasize the importance of interpreting each CBCT image by a trained radiologist. It also alerts the dental community to be observant about the disease to undertake the right diagnosis and precise treatment planning, and avoidance of unexpected complications.

## 2. Case Report

A 21-year-old male reported to the radiology department for evaluation of the potential site for implant placement [5]. The patient was asymptomatic and medically healthy, and presented no known conditions or medications to report on history. There was no related dental history. His social history indicated the absence of smoking or substance abuse.

**Figure 1:**
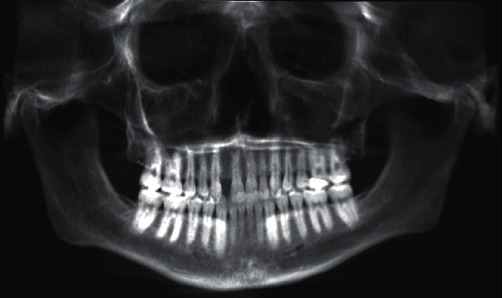
Reconstructed panoramic image.

**Figure 2:**
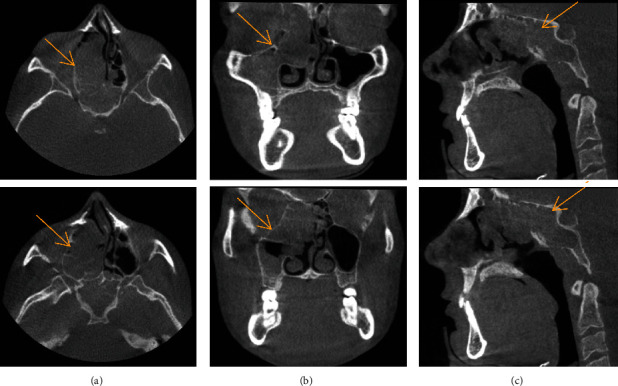
CBCT axial (a), coronal (b), and sagittal (c) images show a large mass occupying the right ethmoid sinus.

On examination, missing right maxillary lateral incisor and third molars were noted (Figure 1). A full field of view cone-beam computed tomography (CBCT) analysis was performed to assess the prospective implant site. The axial slice CBCT images revealed a huge mass with a polypoid shape and soft density that occupied the right ethmoid sinus and sphenoid sinus (Figure 2(a)) [5]. There was a noteworthy enlargement or bulging of the lateral and the medial wall of the right ethmoid sinus. Due to the infiltration or penetration of the lesion to the sphenoid sinus, focal bone erosion of the sphenoid sinus floor was suspected on the sagittal section CBCT images (Figure 2(c)). In addition, this lesion also had progressed into the region of the nose and right maxillary sinus (Figure 2(b)). A significant deviation of the nasal septum was observed. The coronal CBCT images of the right maxillary sinus portrayed significant soft tissue opacification/mucosal thickening involving up to the superior confines of the sinus. In addition, displacement of the inferior medial wall of the right orbit was speculated with no evidence of erosion. There was complete obstruction of the osteomeatal complex on the right side. The middle concha was displaced and was entirely obliterated by the lesion; variation in size of inferior concha was observed as well (Figure 2(b)).

**Figure 3:**
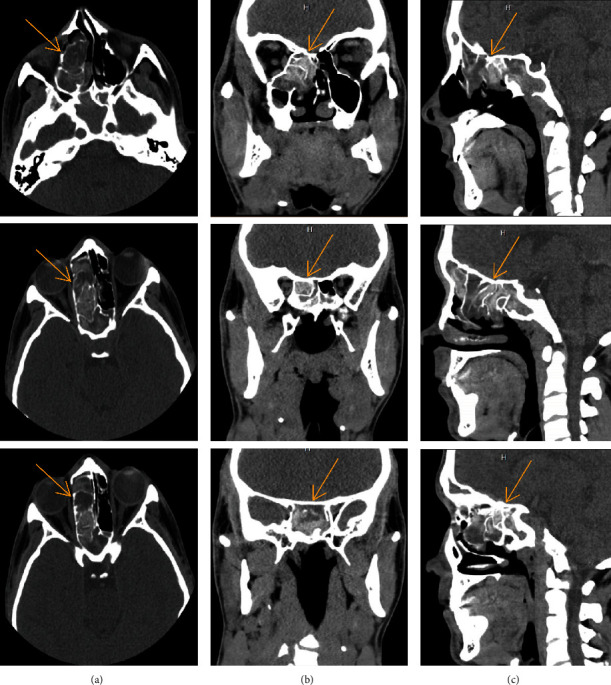
MDCT axial (a), coronal (b), and sagittal (c) images showing opacification and expansion of multiple sinuses with centrally dense inspissated materials.

Contrary to the clinical finding that the patient was asymptomatic, radiographic features were aggressive, which may downgrade the inflammatory course in the differential diagnosis. The patient was directed to an otolaryngologist for further assessment. Further, a non-contrasted multi-detector computed tomography (MDCT) was performed, and the images in all three planes showed inclusive soft tissue opacification in the affected sinuses (Figure 3). Expansion of multiple sinus walls with centrally dense inspissated material with different densities suggested the lesion is more likely to be chronic (Figure 3(a)). The base of the skull and the extraocular muscles were intact, and there was no evidence of this lesion infiltrating into adjacent fat or soft tissue (Figures 3(b), 3(c)) [5]. The internal carotid artery and cavernous sinus were observed within normal limits. The differential radiological diagnosis included sinonasal polyposis, eosinophilic mucin rhinosinusitis, sinus fungal mycetoma, acute rhinosinusitis with complication, and sinonasal squamous cell carcinoma. These radiographic findings were consistent with pathologic findings of chronic invasive fungal sinusitis. Later, the patient was cured with surgical intervention without complications.

## 3. Discussion

This case report presents an incidental finding of fungal sinusitis in a patient who was supposed to undergo a routine dental procedure. Timely radiological diagnosis and appropriate intervention saved the patient from subsequent complications. This case has two significant points of reasoning. First, it stresses the importance of radiological screening before surgical procedures and the application of appropriate imaging for a judicious diagnosis. Second, this study emphasizes the awareness of different life-threatening fungal diseases among dental professionals.

The presentation of fungal colonies in a sinonasal tract is not uncommon and necessarily does not mean an infection. Humans are exposed to a variety of omnipresent fungi and usually do not cause any disease. The immunity of a person plays a significant role in determining the individual's susceptibility to the disease [6, 7]. Depending on the nature of this lesion, it can be a very rapid onset and can cross mucosa to involve vasculature, bone, adjoining soft tissues, orbit, and cranial contents [8]. Timely diagnosis is crucial as it helps treat the patients early to avoid any fatal complications arising due to this. The normal clues of a fungal infection include headaches, nasal congestion, and purulent rhinorrhea [9]. Occasionally it can also show as malaise, fever, bleeding from the nose aka epistaxis, discomfort in the sinus, cough, mucosal ulcerations, and crusting. In serious cases, the presentation will be periorbital swelling, proptosis, and mental status changes [9]. Early detection and appropriate treatment help avoid fatal complications. However, the patient discussed in this case report presented without any symptoms conferred. Hence, the clinician and the radiologist need to affirm that the patient can be asymptomatic. Fungal sinusitis commonly traits complete or partial opacification of the sinus and can portray areas of bone erosion and contiguous soft tissue infiltration. The characteristic location is the maxillary and ethmoid sinuses, but it is observed in the sphenoid sinuses and frontal sinuses, which is rare [10].

Radiographic evaluations are an invaluable tool for early diagnosis and effective management of the disease. CBCT is one of the methods used to assess bony erosion and other osseous changes. Erosion of bone, sinus, and nasal cavity involvement, and mucosal thickening can be found in other diseases, too; however, when found in the immunocompromised patient, one of the differential diagnoses must be invasive fungal sinusitis [11]. Infiltration of soft tissue with fat stranding is poorly recognized in CBCT, and in such cases, multidetector computed tomography (MDCT) or magnetic resonance imaging (MRI) offers superior imaging [11]. In the present report, the patient was asymptomatic and had no relevant medical history. Moderate to severe fungal lesions can precede enlargement and erosion of involved bone spreading to the surrounding cranial and orbital region. It could advance into a potentially deadly consequence if left undiagnosed or untreated. Judicious diagnosis with prompt referral to an otolaryngologist could circumvent grave situations such as loss of sight, and extensive spread of infection leading to unalterable outcomes, even in silent and gradual, asymptomatic development [5].

The hyperattenuating soft-tissue collection could be seen in a non-contrast CT within one or more of the paranasal sinuses. Due to the destructive nature of lesions towards borders of sinuses and expansion beyond the sinus boundaries may resemble malignancy. On contrasted CT, periantral soft tissue and adjacent musculature may enhance depending on the duration of fungal invasion.

Surgical–medical therapy is of paramount importance since it rapidly spreads invasive disease; otherwise, the condition can be fatal. If only the sinus is involved, the prognosis is fair, while it is poor with intracranial involvement. The treatment of choice is radical debridement, anti-fungal therapy, and the treatment of any underlying condition.

With the growing popularity of CBCT and its diagnostic worth, the numbers of dental health professionals utilizing this advanced imaging technology in their practice are sure to increase. Presurgical implant planning and orthodontic and prosthodontic evaluations often include paranasal sinuses; however, the majority of these scans are not evaluated entirely. It is desirable that all the CBCT images are read by a trained oral and maxillofacial radiologist. According to the American Academy of Oral and Maxillofacial Radiology (AAOMR) executive opinion statement, “It is the practitioners' responsibility to examine the entire image dataset” [12]. Eluding the expert interpretation of acquired images might end up in a lawsuit and other legal complications in some countries. Hence, it is essential that there must be awareness among the clinicians and an effort must be made for evaluating each scan by a radiologist. Because if such findings are diagnosed at an early stage and treated radically, the patient will have a good prognosis.

## Data Availability

This report does not contain any patient identifiable information.
